# 2-(2-Chloro­phen­yl)-5-methyl-1,3-dioxane-5-carboxylic acid

**DOI:** 10.1107/S1600536812025019

**Published:** 2012-06-13

**Authors:** Guo-Kai Jia, Lin Yuan, Min Zhang, Xian-You Yuan

**Affiliations:** a College of Chemistry, Xiangtan University, Xiangtan Hunan 411105, People’s Republic of China; b Department of Biology and Chemistry, Hunan University of Science and Engineering, Yongzhou Hunan 425100, People’s Republic of China

## Abstract

In the title compound, C_12_H_13_ClO_4_, the 1,3-dioxane ring adopts a chair conformation and the 2-chloro­benzene and methyl substituents occupy equatorial sites. The carboxyl group is in an axial inclination. In the crystal, carb­oxy­lic acid inversion dimers linked by pairs of O—H⋯O hydrogen bonds generate *R*_2_^2^(8) loops.

## Related literature

For background to protecting groups, see: He *et al.* (2004 ▶). For related structures, see: Laing *et al.* (1984 ▶); Sun *et al.* (2010 ▶); Wang *et al.* (2010 ▶).
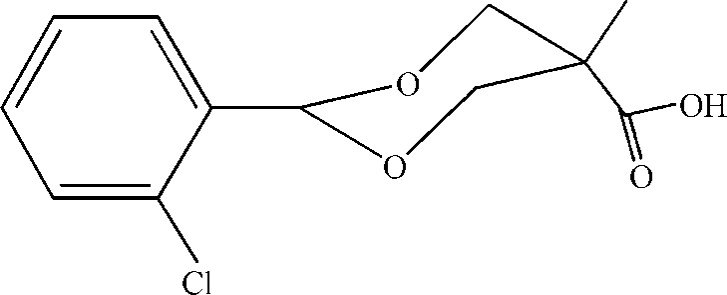


## Experimental

### 

#### Crystal data

C_12_H_13_ClO_4_
*M* *_r_* = 256.67
Monoclinic, 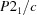

*a* = 9.4452 (3) Å
*b* = 13.9413 (5) Å
*c* = 9.37059 (18) Å
β = 102.145 (2)°
*V* = 1206.28 (6) Å^3^
*Z* = 4
Cu *K*α radiation
μ = 2.83 mm^−1^
*T* = 153 K
0.46 × 0.42 × 0.23 mm

#### Data collection

Agilent Xcalibur Atlas Gemini ultra diffractometer
Absorption correction: multi-scan (*CrysAlis PRO*; Agilent, 2006 ▶) *T*_min_ = 0.356, *T*_max_ = 0.562
5864 measured reflections
2089 independent reflections
1902 reflections with *I* > 2σ(*I*)
*R*_int_ = 0.023
Standard reflections: 0

#### Refinement

*R*[*F* ^2^ > 2σ(*F* ^2^)] = 0.033
*wR*(*F* ^2^) = 0.084
*S* = 1.07
2089 reflections
158 parameters
H atoms treated by a mixture of independent and constrained refinement
Δρ_max_ = 0.33 e Å^−3^
Δρ_min_ = −0.30 e Å^−3^

### 

Data collection: *CrysAlis PRO* (Agilent, 2006 ▶); cell refinement: *CrysAlis PRO*; data reduction: *CrysAlis PRO*; program(s) used to solve structure: *SHELXS97* (Sheldrick, 2008 ▶); program(s) used to refine structure: *SHELXL97* (Sheldrick, 2008 ▶); molecular graphics: *SHELXTL* (Sheldrick, 2008 ▶); software used to prepare material for publication: *SHELXTL*.

## Supplementary Material

Crystal structure: contains datablock(s) I, global. DOI: 10.1107/S1600536812025019/hb6799sup1.cif

Structure factors: contains datablock(s) I. DOI: 10.1107/S1600536812025019/hb6799Isup2.hkl

Supplementary material file. DOI: 10.1107/S1600536812025019/hb6799Isup3.cml

Additional supplementary materials:  crystallographic information; 3D view; checkCIF report

## Figures and Tables

**Table 1:** Hydrogen-bond geometry (Å, °)

*D*—H⋯*A*	*D*—H	H⋯*A*	*D*⋯*A*	*D*—H⋯*A*
O4—H4*B*⋯O3^i^	0.72 (2)	1.92 (2)	2.6323 (18)	170 (3)

Symmetry code: (i) 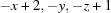.

**Fig. 1.**
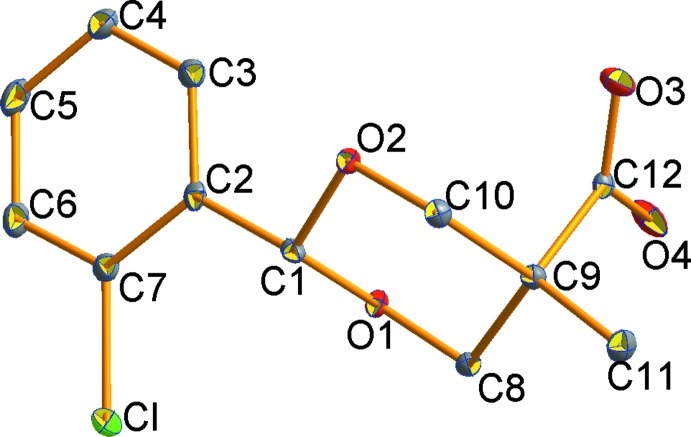
The molecular structure of (I), with 30% probability displacement ellipsoids for non-H atoms.

**Fig. 2.**
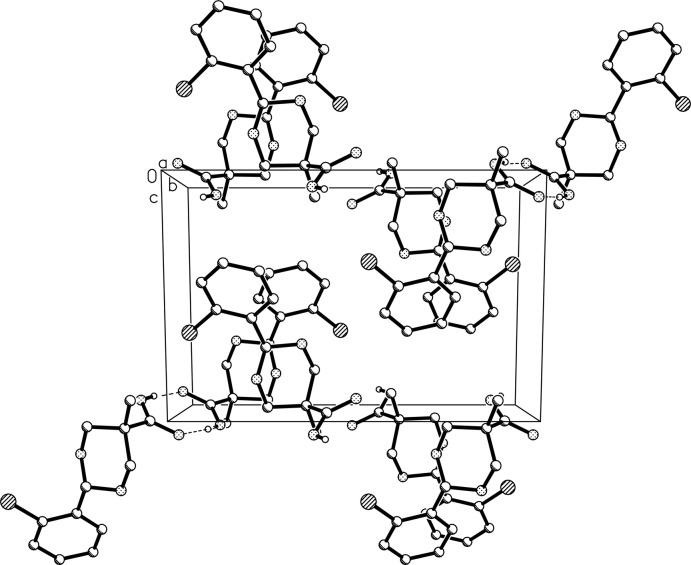
A view of the packing of the title compound

## supplementary crystallographic information

### Comment

The title compound was synthesized to be used as a protection of carbonyl or synthetic intermediate in organic syntheses (He *et al.*, 2004).

In the title compound, C_12_H_13_ClO_4_, the 1,3-dioxane ring adopts a chair conformation and the 2-chlorophenyl substituent occupies an equatorial site (Fig. 1). In the crystal, adjacent molecules are connected by O—H···O hydrogen bonding interactions between the oxygen atoms O_3_ and O_4_ into a dimer (Fig. 2). The crystal structures of some similar 1,3-dioxanes have been reported (Laing *et al.*, 1984; Sun *et al.*, 2010; Wang *et al.*, 2010).

### Experimental

2,2-bis(hydroxymethyl propionic acid (6.7 g, 0.05 mol), 2-chlorobenzaldehyde (7.0 g, 0.05 mol), *N*,*N*-dimethylformamide (30 ml), cyclohexane (15 ml), and *p*-toluenesulfonic acid monohydrate (1 g, 0.005 mol) were heated and stirred at 353 K for 5 h. Diethyl ether (50 ml) and NaHCO3 (0.42 g, 5 mmol) were added to dissolve the residue after DMF and cyclohexane were evaporated under reduced pressure. The organic solution was washed with water (100 ml), and dried with anhydrous sodium sulfate for 3 h. The resulting solution was filtered and evaporated, and the product was recrystallized from ethyl acetate to give 8.3 g of colorless blocks (yield 65%; m.p. 424.2 K).

### Refinement

Carbon-bound H-atoms were placed in calculated positions (C—H 0.95 to 1.00 Å) and were included in the refinement in the riding model approximation, *U*_iso_(H) = 1.2–1.5 *U*_eq_(C). The H-atoms of the hydroxyl groups were placed at calculated positions and then refined as riding; O—H =0.72 Å and *U*_iso_(H) = 1.5 *U*_eq_(O).

### Crystal data

**Table:**

C_12_H_13_ClO_4_	*F*(000) = 536
*M**_r_* = 256.67	*D*_x_ = 1.413 Mg m^−^^3^
Monoclinic, *P*2_1_/*c*	Cu *K*α radiation, λ = 1.54184 Å
*a* = 9.4452 (3) Å	Cell parameters from 5864 reflections
*b* = 13.9413 (5) Å	θ = 4.8–67.0°
*c* = 9.37059 (18) Å	µ = 2.83 mm^−^^1^
β = 102.145 (2)°	*T* = 153 K
*V* = 1206.28 (6) Å^3^	Block, colorless
*Z* = 4	0.46 × 0.42 × 0.23 mm

### Data collection

**Table:**

Agilent Xcalibur Atlas Gemini ultra diffractometer	2089 independent reflections
Radiation source: fine-focus sealed tube	1902 reflections with *I* > 2σ(*I*)
Graphite monochromator	*R*_int_ = 0.023
ω scans	θ_max_ = 67.0°, θ_min_ = 4.8°
Absorption correction: multi-scan (*CrysAlis PRO*; Agilent, 2006)	*h* = −10→11
*T*_min_ = 0.356, *T*_max_ = 0.562	*k* = −16→15
5864 measured reflections	*l* = −10→11

### Refinement

**Table:**

Refinement on *F*^2^	Primary atom site location: structure-invariant direct methods
Least-squares matrix: full	Secondary atom site location: difference Fourier map
*R*[*F*^2^ > 2σ(*F*^2^)] = 0.033	Hydrogen site location: inferred from neighbouring sites
*wR*(*F*^2^) = 0.084	H atoms treated by a mixture of independent and constrained refinement
*S* = 1.07	*w* = 1/[σ^2^(*F*_o_^2^) + (0.0395*P*)^2^ + 0.6772*P*] where *P* = (*F*_o_^2^ + 2*F*_c_^2^)/3
2089 reflections	(Δ/σ)_max_ < 0.001
158 parameters	Δρ_max_ = 0.33 e Å^−^^3^
0 restraints	Δρ_min_ = −0.30 e Å^−^^3^

### Special details

**Table:**

Geometry. All e.s.d.'s (except the e.s.d. in the dihedral angle between two l.s. planes) are estimated using the full covariance matrix. The cell e.s.d.'s are taken into account individually in the estimation of e.s.d.'s in distances, angles and torsion angles; correlations between e.s.d.'s in cell parameters are only used when they are defined by crystal symmetry. An approximate (isotropic) treatment of cell e.s.d.'s is used for estimating e.s.d.'s involving l.s. planes.
Refinement. Refinement of *F*^2^ against ALL reflections. The weighted *R*-factor *wR* and goodness of fit *S* are based on *F*^2^, conventional *R*-factors *R* are based on *F*, with *F* set to zero for negative *F*^2^. The threshold expression of *F*^2^ > σ(*F*^2^) is used only for calculating *R*-factors(gt) *etc*. and is not relevant to the choice of reflections for refinement. *R*-factors based on *F*^2^ are statistically about twice as large as those based on *F*, and *R*- factors based on ALL data will be even larger.

### Fractional atomic coordinates and isotropic or equivalent isotropic displacement parameters (Å^2^)

**Table:**

	*x*	*y*	*z*	*U*_iso_*/*U*_eq_	
Cl	0.64679 (5)	0.45963 (3)	0.15200 (5)	0.02559 (15)	
O1	0.83817 (12)	0.26839 (8)	0.27910 (12)	0.0143 (3)	
C9	0.95610 (17)	0.13397 (12)	0.18663 (17)	0.0139 (3)	
C8	0.96031 (17)	0.24125 (12)	0.22005 (18)	0.0149 (3)	
H8A	1.0508	0.2569	0.2909	0.018*	
H8B	0.9596	0.2779	0.1294	0.018*	
C10	0.80794 (17)	0.11189 (12)	0.08981 (17)	0.0147 (4)	
H10A	0.8003	0.1430	−0.0065	0.018*	
H10B	0.7978	0.0418	0.0741	0.018*	
C7	0.54853 (18)	0.37904 (12)	0.23610 (19)	0.0193 (4)	
C3	0.50347 (18)	0.22009 (12)	0.31020 (18)	0.0181 (4)	
H3	0.5253	0.1535	0.3151	0.022*	
C5	0.3595 (2)	0.35127 (14)	0.3641 (2)	0.0296 (5)	
H5	0.2832	0.3748	0.4062	0.036*	
C4	0.39209 (19)	0.25432 (14)	0.3718 (2)	0.0233 (4)	
H4	0.3385	0.2115	0.4190	0.028*	
C6	0.43677 (19)	0.41421 (14)	0.2957 (2)	0.0275 (4)	
H6	0.4135	0.4806	0.2897	0.033*	
C1	0.70735 (17)	0.24594 (11)	0.17837 (18)	0.0141 (3)	
H1	0.7062	0.2786	0.0831	0.017*	
O2	0.69428 (12)	0.14595 (8)	0.15653 (12)	0.0144 (3)	
C2	0.58361 (17)	0.28173 (12)	0.24141 (18)	0.0153 (3)	
O4	1.05941 (15)	0.10910 (10)	0.43964 (14)	0.0264 (3)	
O3	0.91722 (14)	−0.00573 (9)	0.32146 (14)	0.0271 (3)	
C12	0.97688 (17)	0.07531 (12)	0.32671 (17)	0.0138 (3)	
C11	1.07850 (19)	0.10862 (13)	0.10860 (19)	0.0202 (4)	
H11A	1.1703	0.1340	0.1645	0.030*	
H11B	1.0580	0.1369	0.0107	0.030*	
H11C	1.0854	0.0388	0.1008	0.030*	
H4B	1.073 (2)	0.0772 (17)	0.502 (3)	0.030*	

### Atomic displacement parameters (Å^2^)

**Table:**

	*U*^11^	*U*^22^	*U*^33^	*U*^12^	*U*^13^	*U*^23^
Cl	0.0236 (2)	0.0145 (2)	0.0385 (3)	0.00062 (17)	0.00616 (19)	0.00353 (18)
O1	0.0115 (6)	0.0153 (6)	0.0155 (6)	0.0004 (5)	0.0014 (4)	−0.0026 (4)
C9	0.0145 (8)	0.0154 (8)	0.0122 (8)	0.0008 (7)	0.0038 (6)	0.0004 (6)
C8	0.0143 (8)	0.0152 (8)	0.0159 (8)	0.0003 (7)	0.0046 (6)	0.0015 (7)
C10	0.0171 (8)	0.0154 (8)	0.0117 (8)	0.0013 (7)	0.0034 (6)	−0.0018 (6)
C7	0.0146 (8)	0.0167 (8)	0.0245 (9)	−0.0009 (7)	−0.0010 (7)	0.0001 (7)
C3	0.0170 (8)	0.0169 (8)	0.0188 (8)	−0.0004 (7)	0.0000 (7)	−0.0010 (7)
C5	0.0179 (9)	0.0291 (11)	0.0442 (12)	0.0047 (8)	0.0118 (8)	−0.0053 (9)
C4	0.0162 (9)	0.0256 (10)	0.0283 (10)	−0.0016 (8)	0.0052 (7)	−0.0016 (8)
C6	0.0196 (9)	0.0185 (9)	0.0442 (12)	0.0057 (8)	0.0062 (8)	−0.0027 (8)
C1	0.0147 (8)	0.0111 (8)	0.0148 (8)	−0.0009 (7)	−0.0010 (6)	0.0002 (6)
O2	0.0135 (6)	0.0126 (6)	0.0172 (6)	0.0000 (5)	0.0032 (4)	−0.0029 (4)
C2	0.0119 (8)	0.0166 (8)	0.0152 (8)	0.0010 (7)	−0.0025 (6)	−0.0025 (7)
O4	0.0412 (8)	0.0197 (7)	0.0134 (6)	0.0008 (6)	−0.0053 (6)	0.0030 (5)
O3	0.0298 (7)	0.0195 (7)	0.0278 (7)	−0.0068 (6)	−0.0038 (6)	0.0077 (5)
C12	0.0129 (8)	0.0138 (8)	0.0149 (8)	0.0028 (7)	0.0038 (6)	−0.0015 (6)
C11	0.0201 (9)	0.0241 (9)	0.0183 (9)	0.0028 (7)	0.0081 (7)	−0.0014 (7)

### Geometric parameters (Å, º)

**Table:**

Cl—C7	1.7472 (18)	C3—H3	0.9500
O1—C1	1.4230 (19)	C5—C6	1.382 (3)
O1—C8	1.4314 (18)	C5—C4	1.385 (3)
C9—C12	1.524 (2)	C5—H5	0.9500
C9—C8	1.527 (2)	C4—H4	0.9500
C9—C10	1.530 (2)	C6—H6	0.9500
C9—C11	1.534 (2)	C1—O2	1.4106 (19)
C8—H8A	0.9900	C1—C2	1.501 (2)
C8—H8B	0.9900	C1—H1	1.0000
C10—O2	1.4322 (19)	O4—C12	1.266 (2)
C10—H10A	0.9900	O4—H4B	0.72 (2)
C10—H10B	0.9900	O3—C12	1.259 (2)
C7—C6	1.384 (2)	C11—H11A	0.9800
C7—C2	1.395 (2)	C11—H11B	0.9800
C3—C4	1.387 (2)	C11—H11C	0.9800
C3—C2	1.390 (2)		
			
C1—O1—C8	110.09 (12)	C4—C5—H5	119.6
C12—C9—C8	110.85 (13)	C5—C4—C3	119.60 (17)
C12—C9—C10	109.80 (13)	C5—C4—H4	120.2
C8—C9—C10	107.44 (13)	C3—C4—H4	120.2
C12—C9—C11	108.28 (13)	C5—C6—C7	118.96 (17)
C8—C9—C11	109.50 (13)	C5—C6—H6	120.5
C10—C9—C11	110.98 (13)	C7—C6—H6	120.5
O1—C8—C9	110.48 (13)	O2—C1—O1	110.54 (12)
O1—C8—H8A	109.6	O2—C1—C2	109.51 (13)
C9—C8—H8A	109.6	O1—C1—C2	107.77 (13)
O1—C8—H8B	109.6	O2—C1—H1	109.7
C9—C8—H8B	109.6	O1—C1—H1	109.7
H8A—C8—H8B	108.1	C2—C1—H1	109.7
O2—C10—C9	110.51 (12)	C1—O2—C10	109.89 (12)
O2—C10—H10A	109.5	C3—C2—C7	117.99 (15)
C9—C10—H10A	109.5	C3—C2—C1	121.46 (15)
O2—C10—H10B	109.5	C7—C2—C1	120.52 (15)
C9—C10—H10B	109.5	C12—O4—H4B	114.9 (19)
H10A—C10—H10B	108.1	O3—C12—O4	123.95 (15)
C6—C7—C2	121.73 (16)	O3—C12—C9	118.20 (14)
C6—C7—Cl	118.49 (14)	O4—C12—C9	117.75 (15)
C2—C7—Cl	119.78 (13)	C9—C11—H11A	109.5
C4—C3—C2	121.00 (16)	C9—C11—H11B	109.5
C4—C3—H3	119.5	H11A—C11—H11B	109.5
C2—C3—H3	119.5	C9—C11—H11C	109.5
C6—C5—C4	120.71 (17)	H11A—C11—H11C	109.5
C6—C5—H5	119.6	H11B—C11—H11C	109.5
			
C1—O1—C8—C9	−59.01 (16)	C4—C3—C2—C7	−0.1 (2)
C12—C9—C8—O1	−66.61 (16)	C4—C3—C2—C1	178.11 (15)
C10—C9—C8—O1	53.36 (16)	C6—C7—C2—C3	−0.6 (3)
C11—C9—C8—O1	173.98 (13)	Cl—C7—C2—C3	179.55 (12)
C12—C9—C10—O2	67.01 (16)	C6—C7—C2—C1	−178.79 (16)
C8—C9—C10—O2	−53.64 (16)	Cl—C7—C2—C1	1.4 (2)
C11—C9—C10—O2	−173.32 (13)	O2—C1—C2—C3	19.6 (2)
C6—C5—C4—C3	0.0 (3)	O1—C1—C2—C3	−100.69 (17)
C2—C3—C4—C5	0.4 (3)	O2—C1—C2—C7	−162.29 (14)
C4—C5—C6—C7	−0.6 (3)	O1—C1—C2—C7	77.43 (18)
C2—C7—C6—C5	1.0 (3)	C8—C9—C12—O3	149.70 (15)
Cl—C7—C6—C5	−179.21 (15)	C10—C9—C12—O3	31.1 (2)
C8—O1—C1—O2	64.06 (15)	C11—C9—C12—O3	−90.17 (18)
C8—O1—C1—C2	−176.31 (12)	C8—C9—C12—O4	−33.9 (2)
O1—C1—O2—C10	−64.23 (15)	C10—C9—C12—O4	−152.45 (14)
C2—C1—O2—C10	177.19 (12)	C11—C9—C12—O4	86.24 (18)
C9—C10—O2—C1	59.63 (16)		

### Hydrogen-bond geometry (Å, º)

**Table:**

*D*—H···*A*	*D*—H	H···*A*	*D*···*A*	*D*—H···*A*
O4—H4*B*···O3^i^	0.72 (2)	1.92 (2)	2.6323 (18)	170 (3)

Symmetry code: (i) −*x*+2, −*y*, −*z*+1.
